# Shedding Light on Protein Folding, Structural and Functional Dynamics by Single Molecule Studies

**DOI:** 10.3390/molecules191219407

**Published:** 2014-11-25

**Authors:** Krutika Bavishi, Nikos S. Hatzakis

**Affiliations:** 1 Plant Biochemistry Laboratory, Department of Plant and Environmental Sciences, Center for Synthetic Biology “bioSYNergy”, Villum Research Center “Plant Plasticity”, University of Copenhagen, Thorvaldsenvej 40, DK-1871 Frederiksberg C, Denmark; E-Mail: kru@plen.ku.dk; 2 Bio-Nanotechnology Laboratory, Department of Chemistry, Nano-Science Center, Lundbeck Foundation Center Biomembranes in Nanomedicine, University of Copenhagen, 2100 Copenhagen, Denmark

**Keywords:** single molecules, conformational dynamics, single molecule FRET, free energy landscape, protein folding, allosteric regulation

## Abstract

The advent of advanced single molecule measurements unveiled a great wealth of dynamic information revolutionizing our understanding of protein dynamics and behavior in ways unattainable by conventional bulk assays. Equipped with the ability to record distribution of behaviors rather than the mean property of a population, single molecule measurements offer observation and quantification of the abundance, lifetime and function of multiple protein states. They also permit the direct observation of the transient and rarely populated intermediates in the energy landscape that are typically averaged out in non-synchronized ensemble measurements. Single molecule studies have thus provided novel insights about how the dynamic sampling of the free energy landscape dictates all aspects of protein behavior; from its folding to function. Here we will survey some of the state of the art contributions in deciphering mechanisms that underlie protein folding, structural and functional dynamics by single molecule fluorescence microscopy techniques. We will discuss a few selected examples highlighting the power of the emerging techniques and finally discuss the future improvements and directions.

## 1. Introduction

The arrival of single molecule (SM) techniques to study protein behavior in the last 50 years has unveiled a great wealth of dynamic information—unattainable by conventional averaging biochemical measurements—and provided new insights in the complex tapestry of protein dynamics, function [1,2,3,4,5,6,7,8] and regulation [9]. Single molecule studies offer the direct observation of heterogeneities rather than the average biophysical property from which they can be inferred [10,11,12,13,14,15]. By doing so they offer a unique set of properties all of which remain masked in classical biochemical assays due to averaging of a large number of unsynchronized molecules; firstly they provide the complete distribution of behaviors of the molecular entities in a population, as opposed to only their average behavior. Secondly they allow direct observation of transient intermediates and rare sampled states that remain masked in conventional ensemble kinetics. Thirdly they discriminate static and dynamic heterogeneities within a population. SM measurements have thus unveiled the existence of multiple, and in some cases rarely sampled, functional states [11,16,17,18,19] confirming that proteins do not necessarily reside in the minimum energy of the ground states but may continuously explore the energy landscape while maintaining a native structure, and provided clues on how the sampling of a dynamic spectrum of functional states underlies signaling emergence [20]. Importantly SM studies provide evidence for the anticipated correlations [21,22] of the shape of the rugged energy landscape to all aspects of proteins, from folding to dynamics and function.

Single molecule studies have thus evolved from the initial proof of concept experiments to a guiding principle addressing the needs—and often resolving disputed mechanisms—of biological sciences. The two principal approaches [7,23,24] for observing single molecules rely on micromechanical manipulations and fluorescence spectroscopy and recently their combination [25]. Micromechanical manipulation involves optical and magnetic tweezers [26,27,28,29] as well as atomic force microscopy [30,31,32,33,34], while fluorescence measurements involves polarization, lifetime, particle tracking [3,35,36,37], FRET [7,38] and intensity measurement [7,24,39,40,41,42,43]. Realizing that SM studies has extended to practically all fields of modern biology, furnishing a detailed description of all single molecule results seems rather arduous, so here we shall confine ourselves to a unique set of single molecule methodology, that of fluorescence spectroscopy and the contributions it has made in deciphering mechanisms that underlie protein folding, structural and functional dynamics. This review is organized as follows. We will start with a short description of methodologies to observe protein dynamics. We will then discuss some daunting questions and insights attained by the emerging single molecule techniques on the dynamics that define the folding of biomolecules and underlie their function. The next section focuses on the SM insights on functional dynamics of enzymes. This will be followed up by our concluding remarks and a listing of potentially interesting aspects to watch for in the future.

## 2. Single Molecule Insights into Protein Structural and Functional Dynamics

### 2.1. Methods to Directly Observe Protein Dynamics of Protein Folding and Conformational Sampling

**Figure 1:**
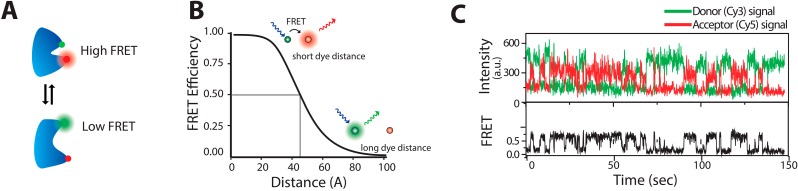
smFRET description (**A**) A biomolecule undergoing time dependent conformations motions changing the inter dye distance and hence the FRET readout; (**B**) Diagram of FRET efficiency, E, as a function of inter-dye distance (R) for a R_0_ = 45 Å. The donor fluorophore when excited with laser either fluoresces or transfers a part of its energy to an acceptor fluorophore, depending on their distance. At distance equal to the Förster radius R = R_0_, E = 0.5. FRET changes are higher at distance variations around the Förster radius; (**C**) Typical time trace of single molecules (here DNA molecules) undergoing conformational fluctuations between two states recorded by fluorescence microscopy. Top trace shows the anticorrelated time traces of donor (green) and acceptor (red) molecules. Bottom trace shows the corresponding E_FRET_. (Figure 1C adapted with permission from [47].)

Deciphering the dynamics of protein that underlie its folding and function has been a central interest of multiple scientific groups [13,44,45,46,47]. The key methods developed for that are Fluorescence Resonance Energy Transfer (FRET) and fluorescence quenching, and have been reviewed elsewhere [1,5,6]. FRET was first used by Stryer and Haugland as a spectrometric ruler to probe distance information [48]. FRET involves a non-radiative transfer of energy from an excited donor fluorophore to a nearby acceptor fluorophore with overlapping spectra and optimal orientation of their transition dipoles. This causes a decrease in the donor fluorescence emission while increasing the fluorescence of the acceptor. The efficiency of the energy transfer (E) can be obtained by determining the ratio of acceptor intensity to total emission intensity. Forster provided the relationship between the energy transfer rate and the donor acceptor distance as E = 1/(1 + (R/R_0_)^6^), where R is the inter-dye distance, and R_0_ is the Förster radius at which E = 50%, see Figure 1. Recording FRET changes at the single molecule level allows observations of conformational fluctuations that occur within the temporal resolution of the instrumentation setup—usually microseconds to seconds.

Single Molecule FRET (smFRET) is carried out primarily in a confocal or wide field microscopy setup [23]. In the confocal microscope, a laser beam is focused on a diffraction limited spot by a high numerical aperture objective lens. A high signal-to-noise ratio is achieved by the insertion of pinholes in the excitation path and in front of the detector, which allow point illumination and block the out-of-focus light respectively. As very low concentrations of fluorescently labeled samples are utilized, single molecules diffusing in the confocal volume can be resolved. Confocal setups are ideal for detection of both freely diffusing and surface immobilized molecules. Using fast and sensitive point detectors such as Avalanche photodiodes (APD) provides up to picosecond time resolution, albeit in a sequential manner hence increasing acquisition time and providing low throughput. Total Internal Reflection microscopy (TIRF) is the workhorse for wide field detection method. It is based on the phenomenon of total internal reflection which generates an evanescent field near the surface (□100 nm). The exponential decay of the field intensity with increasing distance from the glass-water interface results in selective sample illumination and low background signal. Appended to a Charged Coupled Device (CCD) camera, allows parallel detection of several immobilized molecules providing adequate statistics, however with a lower temporal resolution in the submillisecond range.

smFRET is undoubtedly the most general and adaptable among many single molecule fluorescence techniques for biology and is ideally suited to directly record real time distance fluctuations in the range of 2-8 nm. It is however insensitive to shorter ranges of fluctuations that often occur in proteins and are physiologically significant. Recent developments allowed for the first time the observation of conformational dynamics with subnanometer sensitivity. Utilizing single molecule self-quenching of tetramethylrhodamines by the group of Taekjip Ha probed the 0.5 nm conformational motions of an ADP-sensing protein [49].

The techniques discussed above offer an indispensable view of proteins; from directly witnessing and deciphering the mechanisms of folding of proteins to their native state(s) to the observation of conformational dynamics that underlie function. Selected publications and reviews are discussed below.

#### 2.1.1. Single Molecule Insights into Protein Folding Mechanisms

Understanding how the primary amino sequence of the polypeptide chain dictates a unique native structure and the nature of folding pathway that leads to it, has eluded scientists for over 50 years [21,50,51,52,53]. The classical theory of protein folding posits a smooth, deterministic folding pathway having a discrete number of intermediate structures [54,55]. The currently most convincing model of protein folding is that of the “folding funnel” based on the concept of energy landscape. The energy landscape defines the thermodynamic and kinetic parameters that underlie the relative distribution of the conformational states and the energy barriers separating them respectively [56,57]. The folding “funnel” is proposed to be rugged, over which a polypeptide could follow multiple pathways and stochastically sample a plethora of intermediates that funnel the molecular fluctuations *en route* to the native state [53,58,59]. The existence of multiple pathways and the conformational sampling of transient intermediates can be described by theoretical models of protein folding and computer simulations but is masked in conventional measurements that report the average property of a large ensemble of unsynchronized molecules. Single molecule studies with the advantage of directly probing multiple subpopulations and stochastic dynamics in a heterogeneous ensemble are emerging as new and powerful tools in deciphering the mechanism of these complex processes. Protein folding insights obtained by single molecule studies have been extensively reviewed elsewhere [1,60,61,62,63]. A few selected examples shall be discussed in the following section.

Pioneering SM fluorescence-based studies on protein unfolding were performed on dual labeled, freely diffusing molecules in equilibrium conditions. Deniz *et al*. utilized smFRET to characterize the unfolding of Chymotrypsin Inhibitor (CI2) under equilibrium conditions [64]. A TMR/Cy5 labeled CI2 was subjected to different concentrations of the denaturant guanidinium chloride and analyzed by FCS allowed the observation of the existence of two populations—folded and unfolded—and how their relative distributions are altered by different denaturant concentrations and more importantly by destabilizing mutations. FRET efficiency histograms revealed that at close to native (low) concentrations of GdmCl, CI2 appeared as a single predominantly folded species with a high FRET efficiency. Increasing the denaturant concentration to intermediate levels split the unimodal FRET distribution to having a second off-peak with low FRET efficiency indicating the equilibrium between the folded and unfolded states. When increasing further the GdmCl concentration the high FRET peak disappeared, which indicated the collapse of the ordered conformation to an unfolded one. This confirmed the presence of a simple two state folding pathway for CI2, previously suggested by bulk studies. Similarly, a two-state folding pathway was observed for the cold shock protein from the bacterium *Thermotoga maritima* (CspTm) [65] and RNase H [66] by SM measurements in equilibrium conditions. Interestingly, a combination of smFRET and NMR approach on the gradual unfolding of a slow two state folder SH3, suggests that folding mechanisms under biological conditions could differ from those deduced by experiments conducted in denaturing conditions due to plasticity of the folding landscapes [67].

Protein unfolding studies conducted under freely diffusing conditions could mask the existence of subpopulations that are sparsely populated at equilibrium. To observe folding kinetics under non-equilibrium conditions and study the possible existence of subpopulations in the folding of CspTm Lipman *et al*. [68] employed a microfluidic laminar flow mixer device [69] coupled to a confocal microscope. While diffusing through the confocal volume proteins were subjected to an abrupt reduction in denaturant concentration that favored the folded state. However, they identified only two major conformations, confirming earlier studies. Recently a low-cost microfluidic mixing device based on hydrodynamic focusing and diffusing mixing has been developed by the group of Schuler [70]. Using this setup they measured smFRET on unfolding of the B-domain of protein A and the conformational dynamics of the pore forming toxin ClyA, by mixing of the denaturant GdmCl and detergent *n*-dodecyl-β-D-maltopyranoside (DDM), respectively. This setup offers measurement of single molecule kinetics at the timescales of milliseconds to minutes.

**Figure 2:**
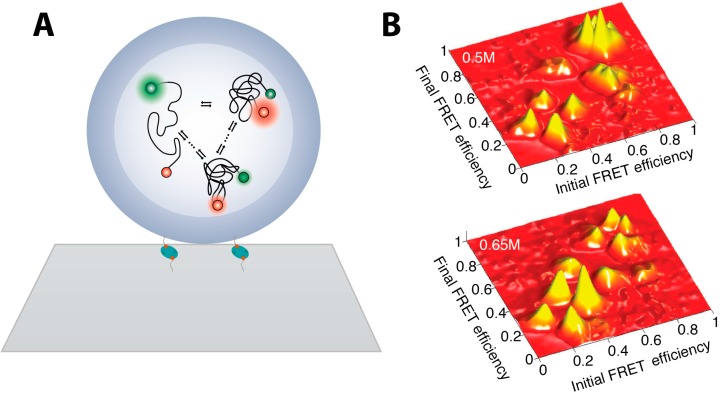
smFRET studies of adenylate kinase enzymes folding (**A**) Individual AK enzymes, double labeled for smFRET measurements, were encapsulated inside liposomes minimizing interaction with hard matter allowing unbiased measurements of the folding landscape. Liposomes are tethered on glass microscope surface via biotin-avidin interaction; (**B**) Transition density plots showing the preferred transition pathway of folding to depend on denaturant concentrations. As the denaturant concentration increases more transition between lower FRET states occur and the fraction of sequential—between states of similar FRET intensity—increases (Figure 2 is adapted from [74] with permission).

In order to attain long trajectories of individual molecule and obtain ample data for statistical analysis, immobilization strategies were invoked. However, direct immobilizing proteins on surface may induce non-native surface interactions that may interfere with single molecule studies [2,71,72]. Seminal work on employing star-shaped polymers to minimize non-specific interactions with the glass surface showed that proper surface passivation may not affect dynamics [66]. This paved the way for the wide implementation of passivated surfaces with PEG polymers and the use of biotin-avidin interaction to immobilize biomolecules. Lately a very promising immobilization strategy has been developed which involves encapsulation of proteins in lipid vesicles that are tethered on the microscope slide surface via e.g., avidin biotin interactions, see Figure 2A. This approach allows spatial localization of protein under investigation minimizing interactions with non-native environment. Utilizing this technique for unfolding studies on adenylate kinase (AK), led to the observation of multiple folding pathways and the existence of multiple, small partial folding/unfolding jumps. This indicated the presence of small local traps on the rugged free energy landscape [73]. This strategy was further exploited by Pirchi *et al*. [74] which has identified the existence of six metastable states in the multidimensional folding landscape of AK. Using a large number of equilibrium smFRET trajectories of AK they have directly observed the pathway that connects these states and its rerouting by changes in denaturant concentration. They also recorded the rates of their interconversion in the folding process as shown in Figure 2B. These data allowed sketching of a multiple state energy landscape, while hinting towards a “foldon” hypothesis [75].

In the quest of attaining information on distribution of folding pathways, the group of Eaton has employed a single molecular FRET approach to determine the average transition path times of a fast and slow folding protein [76] as well as the dynamics of barrier crossing [45]. They demonstrated that despite a 10,000-fold difference in their folding rate coefficients, the transition path times differ only by a factor of five. Hence the slow and the fast folding proteins would take almost approximately similar time to fold when the folding process would occur [76].

Single molecule studies have recently expanded in dissecting the mechanism of folding and function of Intrinsically Disordered Proteins (IDP). IDPs lack, partially or completely, an ordered 3D structure and are involved in a spectrum of diseased states such as cancer, cardiovascular and neurodegenerative disorders and diabetes [77,78,79]. While their inherent dynamic nature is challenging to be studied by conventional bulk methods the ability of SM methods to directly detect and quantify fleeting intermediates has an especially important take-away in IDP. The greatest advantage of SM has been in investigating amyloidogenic IDPs where the low protein concentration requirement of the SM studies minimizes the formation of higher order oligomers and allows studies of the folding intermediates. smFRET studies have been extensively employed for deciphering the folding mechanisms and conformational sampling of multiple IDPs such as the NM-region of the yeast prion protein Sup 35 [80], α-Synuclein [81,82,83,84], islet amyloid polypeptide (IAPP) [85] and Tau proteins [86]. These studies have highlighted the role of environmental conditions such as presence of denaturants, membranes or osmolytes on the structural fluctuations and dynamics in IDPs. In addition, manifestation of allostery has been demonstrated in a molecular hub IDP, the adenovirus early region 1A (E1A) oncoprotein [87]. Similarly the group of Schuler has investigated the effects of temperature, pH and crowding agents on the conformational collapse of unfolded proteins and IDPs [46,88,89]. Using a combination of smFRET, theory and simulations they provided new, often unexpected, insights on the collapsing protein behavior. Their studies showed that the temperature-induced collapse is a common feature of multiple IDPs with different sequences [89]. They furthermore revealed that in addition to hydrophobic effects, the temperature-dependent solvation free energies of the relevant amino acids have a dominant contribution in the collapse. Similarly their studies in the presence of crowders revealed IDP compaction to depend not only on the concentrations but also on the size of the crowder [46]. This unexpected behavior, based on the theory from scaled-particle, was explained quantitatively by taking into account the polymeric nature of the crowder and the protein. A very recent and exhaustive review enlisting the wealth of work on the application of single molecule studies to IDPs has been published [90]. Probably the most exploited field by smFRET involves measurements of conformational dynamics of folded proteins and biomolecules. In the next section, we will focus on how SM measurements could decipher the highly debated role of conformational motions to the catalysis step.

#### 2.1.2. Single Molecule Insights into the Role of Protein Conformational Dynamics to Function

Proteins are conformationally inhomogeneous and inherently dynamic; a phenotype that stems from the stochastic search of intermediates during folding and extends to the sampling of the conformational space in the native state. Several review papers addressing this fundamental attribute of proteins have been published [21,22,91,92]. While it is in general well appreciated that these dynamics and exploration of the conformational space are important attributes of protein function, deciphering how exactly these dynamics control protein function has eluded scientists for over 30 years [93] and has been the central quest of multiple research groups [91,94,95,96,97]. In fact, it is currently being disputed whether protein dynamics do catalyze the chemical step [98]. Single molecule structural studies are becoming an indispensable tool in the quest of decrypting the potential role of protein dynamics in function. In this section, after briefly outlining the current debate on the direct coupling of conformational dynamics to the catalysis step, we will show how emerging single molecule techniques could potentially provide solutions to this debate by directly and synchronously recordings of protein conformation and function. In Section 2.2 we will discuss the only methods to directly observe individual catalytic cycles but not the conformational dynamics that potentially underlie them.

Pivotal work from the group of Kern aimed to realize if enzyme conformational motions are random or pre-encoded to follow a pathway optimized for the chemical step [99]. They utilized adenylate kinase which has two “substrate lids” that within the catalytic cycle undergo a transition from the open to the close conformations. Combining smFRET readout with dynamic simulation and NMR studies revealed that AK exists in equilibrium between an open and closed state. Importantly they directly observed protein conformational motions to occur in similar time scales with the catalysis step, as calculated by ensemble averaging kinetic measurements. Their data suggested that protein motions are not random but deterministic and directed by the energy landscape; the fast time scale (picoseconds to nanoseconds) hinge domain motions collectively drive the slow large domain motions (microseconds to milliseconds) that are anticipated to be catalytically important. They thus proposed that the hierarchy of protein dynamics in space and time arises from the protein structure encoded by the amino-acid sequence and is ultimately connected to enzyme function. This claim however; of the direct coupling between conformational dynamics and chemical kinetics is now being heavily challenged [100,101]. Deciphering such conundrums may require direct and synchronous observation both conformational motions and function that to date can only be attained by single molecule studies.

**Figure 3:**
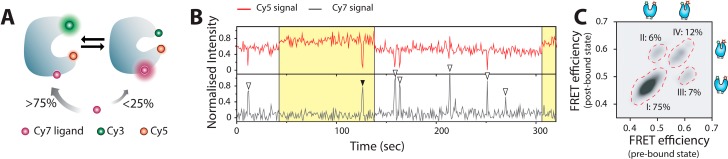
Direct and synchronous observation of protein conformational dynamics and ligand binding on an individual Maltose Binding Protein to decipher the mechanism of action. (**A**) Cartoon representation depicting the existing equilibrium between an open and closed state and ligand binding preferentially to the open state; (**B**) Three color smFRET traces depicting the binding of Cy7-maltose to an open and partially closed state of MBP as open and solid arrowheads; (**C**) Correlation plot of Cy3-Cy5 FRET efficiencies both at the binding and dissociation points for the binding events of single Cy7-maltose showing the relative distribution of the four possible scenarios; type I, binding to an open state and dissociation from the same state; type II, binding to an open state and dissociation from a partially closed state; type III, binding to a partially closed state and dissociation from an open state; and type IV, binding to a partially closed state followed by dissociation from the same state. (Figure 3 adapted from [102] with permission).

The group of Kim recently attained the direct and synchronous observation of protein conformational dynamics and function of an individual maltose binding protein (MBP) [102]. To do this they performed three-color FRET, by combining a Cy3/Cy5 FRET pair labeled MBP and fluorescently Cy7 tagged ligands as shown in Figure 3. Their results reveled that although capable of binding to both closed and open forms, ligands preferentially interact (>75%) with the closed state and that ligand binding also altered primarily the closing rates of MBP, directly observing for the first time, how structure of the protein controls its function. Later studies revealed that intrinsic opening rates also control ligand dissociation and hence binding affinity of MBP [103]. Their single molecule readout also deciphered whether the mechanism of underlying ligand recognition operates via the “Induced fit” or the “conformational selection” model [9,92].

### 2.2. Methods to Attain Single Molecule Insights in Protein Functional Dynamics

Single molecule functional studies have revolutionized our understanding of how enzymes work. The journey from Rotman’s first attempts [104] to date has been remarkable, with the development and implementation of several methodologies to monitor the activity of individual enzyme molecules. Single particle tracking studies were used to correlate distance information to enzymatic activity and reveal the underlying mechanisms for several enzymes such as kinesin [12], dynein [105] and phospholipases [106,107]. The workhorse of single molecule fluorescence functional assays is a “tag” capable of demonstrating fluctuations in its own fluorescence to resolve the individual catalytic cycles of an enzyme.

**Figure 4:**
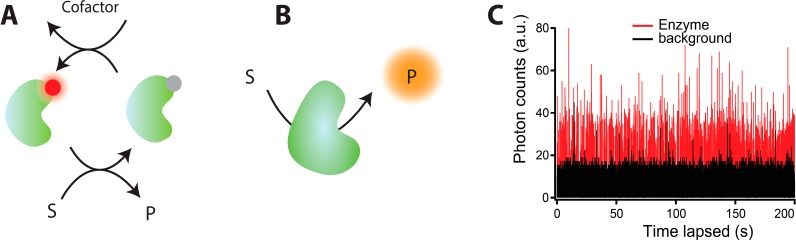
A general description of the methodology to attain single turnover resolution measurements. (**A**) A method exploiting fluorescence property changes within each catalytic cycle of cofactors or covalently attached fluorophores; (**B**) Prefluorescent substrate analogues for prolonged observation of individual catalytic turnovers. The non-fluorescent substrate is converted by the enzyme to the highly fluorescent product, which subsequently diffuses away from the detection volume allowing recordings of individual catalytic cycles. Being limited only by substrate depletion this methods allows recordings of thousands of catalytic cycles for accurate statistical analysis; (**C**) A typical single molecule activity trace of lipase converting a prefluorescent substrate analogue, here CFDA, to highly fluorescent carboxy-fluorescein (red trace). Each spike corresponds to an individual catalytic cycle. The black trace corresponds to the background signal (Figure 4C reprinted with permission from [17]. Copyright (2012) American Chemical Society.)

The initial single turnover resolution experiments utilized the intrinsic fluorescence properties of the flavin cofactors of enzymes [11,108,109], see Figure 4A. As these flavin cofactors cycle between their fluorescent oxidized (“on”) state and non-fluorescent reduced state (“off”), they provide real time information of individual stochastic catalytic cycles. The greatest advantage of this approach was the absence of mutagenesis and site specific labeling while its inherent limitation was the low quantum yield of the cofactors and their tendency to dissociate and bleach limiting the fluorescent trajectories to a few turnovers. Such limitations were surpassed by employing the redox state dependent quenching of a biochemically attached photostable fluorophore on Nitrite reductase from *Aspergillus niger* [110,111]. Within each turnover cycle its copper cofactor sequentially transits from a reduced to the oxidized which in turn efficiently quenches the site-specifically attached fluorophore. However, the credit of revolutionizing single molecule functional assays goes to prefluorescent substrate analogues, which discarded the pre-requisite for enzymes to have fluorescent cofactors or undergo significant structural transitions in order to record functional cycles, see Figure 4B. In this approach, non-fluorescent substrate analogues upon enzymatic reaction are converted to highly fluorescent products generating the stochastic single molecule fluorescence (SMF) trajectories like the one shown in Figure 4C. The superiority of this technique lies in overcoming the issue of photo bleaching, allowing observation of large number of turnovers limited only by substrate depletion, significantly improving statistical analysis. This platform has been applied to a broad and diverse class of enzymes ranging from β-galactosidase [112] to lipases from *Candida antarctica* (CALB) [19], *Thermomyces lanuginosa* (TLL) [17,113], proteases like chymotrypsin [114], and oxidoreductases like Horse radish peroxidase [115] and P450 Oxidoreductase (POR) [16].

Single turnover resolution measurements revealed previously masked (a) time dependent activity fluctuations for enzymes and (b) heterogeneities in activity between seemingly identical enzymes of a population. The observation of the existence of these heterogeneities, have been discussed earlier [39,116]. Here we will provide latest insights and attempts to provide explanations of the mechanistic origin of these heterogeneities. Some of the wide implications of these heterogeneities will then be discussed.

#### 2.2.1. Dynamic Disorder: Sampling of a Few or a Spectrum of Activity States?

Dynamic disorder is the time-dependent activity fluctuations of an enzyme. Initially dynamic disorder was proposed to originate from the existence of a continuous distribution of protein states with different activities, a phenotype that was anticipated to pertain to the majority of enzymes. Latest studies however provided evidence that a limited number of discrete functional states may equally well describe the behavior of multiple enzymes. Here we will outline these studies and their insights. The first direct observation of dynamic disorder was presented by the group of Xie on molecules of cholesterol oxidase by monitoring fluorescence fluctuations of its FAD cofactors [11]. Enzymes were spatially confined in a porous agarose gel which allowed free exchange of its substrates and products. Statistical treatment of SMF trajectories yielded waiting time histograms and autocorrelation functions, which did not fit with a monoexponential function indicating time dependent activity fluctuations. While the activity fluctuations could be fit with a model of two activity states the authors proposed the existence of multiple protein states. Ever since, time dependent activity fluctuation of individual molecules has been found to be representative for most water soluble enzymes studied up till date such as Horse Radish Peroxidase [115], Lipases from *Candida antarctica* (CALB) [19] and *Thermomyces lanuginosua* (TLL) [17,113], β-galactosidase from *E. coli* [112], nitrite reductase [110,111], dihydroorate dehydrogenase [72], hydroxybenzoate hydroxylase [108], and P450 oxidoreductase [16].

We recently extended the observations of dynamic heterogeneities and identified for the first time the existence of discrete functional states that are linked to the enzyme’s conformational states [17]. Using arrays of surface tethered liposomes [117,118,119,120,121,122,123,124,125] we investigated the activity and regulation of a membrane related enzyme, the lipase. Lipases are enzymes that catalyze the enantioselective hydrolysis of esters in solution [126,127,128,129,130] with attractive biotechnological applications [131,132]. TLL is a typical interfacial activated lipase model system that shows low or no activity in solution but in the presence of an interface *i.e.*, membranes the peptide-lid that otherwise blocks its active site is displaced, rendering TLL catalytically active [133]. We tethered TLL on surface immobilized liposomes by a flexible linker and interrogated its activity on prefluorescent substrate analogues by titrating in a progressive and quantitative manner its proximity to its effector, the lipid membrane. Our findings revealed TLL to oscillate between two functional states a highly active one and a practically inactive one. Importantly these functional states correlated with the enzyme major conformational states; the inactive state can be assigned to the closed lid conformation while the highly active state to the open lid conformation.

**Figure 5:**
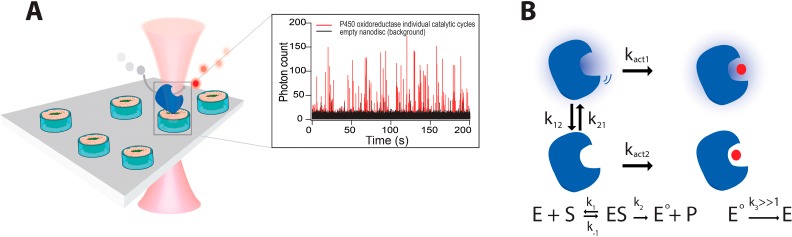
(**A**) First measurements of individual turnover activity on membrane spanning enzymes (POR) on prefluorescent substrate, by using Nanodiscs—nanometer scale membrane patches—to spatially confine them in native like membrane environment; (**B**) Schematic representation of a general two-state model that accurately describes the catalytic behavior of multiple enzymes POR, TLL, CALB, NiR (see in text). The model is an extension of the Michaelis–Menten equation and contains two interconverting functional states with respective activity rates *k_act1_* and *k_act2._* Adapted with permission from [16]. Copyright (2014) American Chemical Society.

To investigate whether the existence of a discrete number of functional states pertains to transmembrane enzymes, we recorded the first single turnover measurement of a membrane-spanning enzyme P450 oxidoreductase (POR). To maintain POR in “native like membrane environment” we employed nanodiscs [134] to reconstitute P450 oxidoreductase (POR) [16]. POR is the obligatory electron donor to all microsomal P450s [135,136] and aberration in its function results in spectrum of diseased states ranging from cortisol deficiency to skeletal dysplasia [137,138,139]. A single cysteine POR variant labeled with Cy5 was reconstituted in DiO labeled Nanodiscs that were subsequently tethered on passivated surfaces, see Figure 5A. Using confocal fluorescence microscopy we recorded individual POR catalytic cycles on a prefluorescent substrate analogue resazurin. Our results revealed POR to sample to two major functional states, a highly active and a practically inactive one in agreement with our finding on TLL [2,9] (see Figure 5B).

To date the existence of dynamic disorder is evident in most enzymes and is intuitively assumed to originate from the enzymes sampling of a spectrum of conformations, each of which has different activity. Proteins are indeed highly dynamic exploring the conformational space and sampling conformations in time scales that range from ps to ms or longer [91,92,140,141,142,143]. The initial single molecule functional studies revealed time dependent activity fluctuations in the same time scale as slow protein conformational motions. It was thus intuitively assumed that these activity fluctuations stemmed from protein conformational fluctuations. In fact even though the activity fluctuations of the pivotal studies on cholesterol oxidase and dihydropholate reductase were sufficiently interpreted by models with two functional states [11,144,145,146] more complex behavior was assumed and multiple activity states models were used [144,145]. This phenotype was adopted by a variety of other single molecule studies proposing enzyme to sample a spectrum of conformational states each with its own activity [23,39,40,116,147].

Increasing evidences support that simpler models with a discrete number of functional states may adequately describe the behavior of enzymes [17,146,148,149,150,151]. Our recent results supported this emerging notion by showing that multiple—though not all—enzymes sample two rather than multiple functional states. We recorded the activity two lipases [17], (one from *Candida antarctica* and the other from *Thermomyces lanuginosua*) and *POR* [16]. We also retreated published data for the nitrite reductase from *Alcaligenes faecalis* [111], bovine α-chymotrypsin [114], and β-galactosidase from *E. coli* [112] in a way covering most of the enzymes where single turnover resolution measurements with high statistics is available. The tetrameric β-galactosidase as expected exhibited more complex behavior. Importantly we found that the behavior of the lipases, nitrite reductase and POR—four out of the five monomeric enzymes—was accurately described by two rather than multiple functional states. The existence of a small number of discrete functional states was also directly observed by pioneer single molecule measurements on lysozyme [148,152] further supporting this to be a generic phenotype underlying the behavior of multiple enzymes.

Along the same lines, recent work by Terentyeva *et al*. highlighted the possible limitations and artifacts that may arise from treating single turnover trajectories and questioned the existence of dynamic disorder for α-chymotrypsin, and possibly for other enzymes. Performing a systematic evaluation of commonly used binning and thresholding methodology of published data on α-chymotrypsin and simulated data they illustrated that the concave shape of the waiting time distribution—widely employed as a hallmark of distribution of functional states—may be due to artifactual data treatment [150,151]. They proposed change point analysis as a more accurate methodology to evaluate data and illustrated α-chymotrypsin to have a single activity over time rather than sampling multiple functional states. Their data signified the difficulties to accurately determine the number of exponentials underlying the waiting time histograms, if the number of exponentials needed for the fit is more than three. To resolve similar issues, Flomenbom *et al.*, developed methodology for filtering noise using numerical algorithm that is based on a general likelihood function [153].

The existence of a discrete number of functional states does not repudiate the inherent protein dynamic behavior. Regulated proteins, ion channels and enzymes often oscillate between active and inactive conformations. Enzymes throughout the evolutionary course may have optimized what is needed for proficient regulation discarding unwanted functional states, maintaining few or two of them with large activity differences. The highly active states(s) of enzymes may emanate from optimized conformational coordinate and dynamics towards what is important for barrier crossing and catalysis [92,154,155]; any improper active site organization or dynamics relevant for catalysis would result on the practically inactive state(s). Importantly the existence of a small discrete number of functional states is in full agreement with the simple two-state models often employed by ensemble studies to describe regulated protein behavior [156,157], bridging ensemble with single molecule measurements.

#### 2.2.2. Static Disorder: Multiple Distinct Folds of the Same Sequence or Chemical Heterogeneities?

Static disorder describes the heterogeneities of activity rates between individuals of a seemingly identical population. The path breaking work on single molecules by Rotman yielded the first hints for the existence of static heterogeneity by proposing that heat inactivation of enzymes occurred in an “all or none” manner [104]. However, it took 30 years of methodological improvement to attain direct observation of static disorder. Xue *et al*. reported up to a four-fold difference in the activities of individual lactate dehydrogenase enzymes lasting for a period of around 2 h [158]. However, the nature of origin of these functional differences has been debated ever since. The two opposing theories propose static heterogeneities to emerge either from chemical heterogeneity or presence of distinct, long lived conformational states. The latter could be a result of different folding patterns for different molecules whereby each could reside in different local minima separated by high energy barriers in the free energy landscape.

Chemical heterogeneities such as proteolytic damages and post translational modifications of the protein were proposed to underlie the observed static disorder in cholesterol oxidase [11] and alkaline phosphatase respectively [159]. The hypothesis of long lived conformational states related disorder was supported by the low throughput measurements on lactate dehydrogenase enzymes [158]. To scale up the number of molecules available for analysis the group of Walt *et al.* developed a high throughput assay utilizing femtoliter size reaction vessels that allowed the parallel readout of the activity of hundreds of individual enzymes [18,160]. The temporal resolution of a few to 10s of seconds averaged out millisecond activity fluctuations and allowed the observation of a broad distribution of activity rates. These were found to originate from a distribution in k_cat_ rather than K_m_. The static heterogeneities were thus attributed to distinct long lived activities of the enzyme, however transitions between such long lived states were not directly observed. Later studies by the same group used short heat pulses to record switching of the β-galactosidases between long lived states of different activities [161]. The authors noted that only 25% of the β-galactosidase enzymes have identical amino acid sequences during the expression highlighting the contribution of chemical heterogeneities in static disorder. The heat dependent transitions between some of the protein states however support the existence of multiple thermodynamically trapped states with different activities in the protein energy landscape. Similarly smFRET measurements directly observed individual ribozymes to fold into multiple distinct native states with different activity [162]. Ribozymes were found to interconvert between these states in ultra-slow time scales (~9% of molecules in 40 min). It is thus becoming increasingly apparent that although some contribution from chemical heterogeneities may exist, static disorder may be a form of ultra-slow dynamic disorder. Static disorder may thus emerge from the inherent capacity of proteins to adopt multiple distinct folds and explore multiple energy minima in the free energy landscape separated by higher energy barriers.

**Figure 6:**
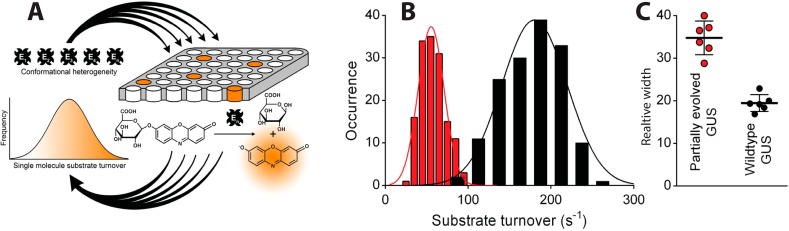
Single molecule insights on the functional diversity of enzymes. (**A**) high density arrays of 62,500 fL reaction chambers to simultaneously interrogate the activity of hundreds of individual enzymes. The nonfluorescent substrate ReG is converted to the highly fluorescent product resorufin that is detected by fluorescence microscopy. The method allows observation of the existence of long lived functional states; (**B**) Widths of distributions of the sampled activity states for the wild type (black) and partially evolved GUVs following in both cases Gaussian distributions, but having variable widths (**C**) Relative widths of distributions (coefficient of variation) of multiple independent experiments showing the activity of the partially evolved GUVs to be significantly more widely distributed as compared the wild type enzyme (reprinted with permission from [164]. Copyright (2014) American Chemical Society.

The manifestation of multiple long lived states that extend into the realm of protein lifetime are expected to have mechanistic and biological significance since evolutionary pressure is required to evolve and maintain them [163]. Indeed recent pivotal work by the group of Gorris provided the first single molecule insights on the functional diversity of evolving enzymes [164]. Using arrays of femtoliter reaction chambers they compared the activity behavior of β-glucuronidase (GUS) and partially evolved GUS variants—called generalists—that retain their wild-type activity but also exhibit promiscuous activity accepting a broad range of other substrates. Their findings revealed significantly more broad long lived activity fluctuations for the partially evolved enzymes as compared to the wild type, see Figure 6. These data provided the first links of widths of activity distributions to increased variant promiscuity supporting the notion that increased widths of activity distributions-and thus functional promiscuity may serve as evolutionary starting points for the functional adaptation of enzymes [165].

## 3. Current Improvements and Future Directions

Great strides have been made in characterizing protein dynamics by single molecule fluorescence studies, however we are still at the tip of the iceberg and further improvements are required to keep in pace with their ever widening scope and applications of biology. There is a growing demand for a progressive evolution and combination of the presently available methodologies while simultaneously delivering novel solutions to combat limitations of the conventional techniques.

Attaining a comprehensive description of protein function *in vivo* requires implementation of powerful new technologies that combine single molecule readout in native-like conditions and parallel screening of biomolecular interactions. The development of high-density microarrays of biomimetic scaffolds such as liposomes [17,73,118,119,120,121,166], nanodiscs [16,167,168] and polymer appended self-assembled nanostructures [169,170,171,172] for single molecule studies could be a novel way to implement screening of biochemical properties, molecular function or protein-effector and membrane interactions. Importantly it allows for the first time single molecule investigation of membrane spanning enzymes [16,117,167,173,174] in their native environment, the membrane. The massive parallel readout (10^3^–10^4^ particles per frame) of such arrays could allow the direct observation of protein native states and quantification of their activity, abundance and importantly their dependence on regulatory inputs. Performing single molecule studies on crude cell extracts [175,176] is also bridging the gap between *in vivo* and *in vitro* platforms. As the native environment is preserved, it is possible to analyze in real time, the assembly, functioning and stoichiometry of macromolecular complexes. The development of methodologies like Photo Activation, Diffusion and Excitation (PhADE) [177] extends the useful concentration range for single molecule fluorescent imaging by at least two orders of magnitude and enables single molecule visualization at physiological concentrations.

A fundamental challenge of performing SM fluorescence experiments is site-specific coupling of the fluorophore (organic dyes, fluorescent proteins, quantum dots *etc.*) without perturbing the native structure and function of the host molecule. Although bioconjugation to specific thiols is the most efficient strategy, labeling of large proteins or those with high cysteine content could be difficult. Moreover, smFRET requires attachment of two fluorophores at specific positions. A possible solution to these is the use of bio-orthogonal chemistry [178,179] such as click chemistry [113,171,180,181,182,183] to tag unnatural amino acids to provide a site specific and non-invasive labeling alternative. Utilization of recent advances of direct site specific bioorthogonal protein labeling in bacteria and cells is expected to improve single molecule studies in live cells [184,185,186].

Further, organic fluorophores are inherently labile and prone to reversible (blinking) or irreversible (bleaching) deactivation that limits their utility and performance. Although oxygen scavengers and triplet state quenchers are routinely added to delay such photophysical phenomena [187] these are poorly soluble and could be potentially harmful to biomolecules and alter the properties of bilayers [188]. The group of Blanchard demonstrated enhanced photostability and reduced blinking of several cyanine dyes by proximal conjugation of “protective agents” such as cyclooctatetraene (COT), 4-nitrobenzyl alcohol (NBA) or Trolox. These “self-healing” dyes could enhance the performance of SMF assays by providing a stable and robust reporter system, extending the observation regime to shorter time scales and potentially bridging gaps between experimental and computational methods [189,190,191]. Similarly Metal Enhanced Fluorescence (MEF), achieved by placing metal nanoparticles in close proximity to organic fluorophores offers enhanced fluorescence intensity and stability and has been successfully employed for single molecule FRET analysis of ribosomes [192]. Development of near infra-red fluorophores [193] and improving the properties of presently available quantum dots and fluorescent proteins will further expand the realm of SMF studies.

Utilizing emerging and currently existing SM techniques to measure structural dynamics will advance our understanding on the full fluctuation spectra of proteins and its interactions with partners. The established alternating-laser excitation (ALEX) methodology that simultaneously reports on structure, dynamics, and stoichiometries of fluorophores, by directly exciting the donor and the acceptor fluorophores of a FRET pair in an alternate fashion, is an invaluable tool in this quest [194,195]. Similarly the evolution of the conventional single pair FRET to multicolor FRET enables dissecting complex biological phenomena by allowing simultaneous observation of multiple parameters. Three and four color-FRET, combined with ALEX can evaluate three or six pairwise distances [196,197] allowing studies of dynamic multicomponent biomolecules. The group of Seidel recently developed a FRET-restrained high-precision structural modeling that explicitly accounts for the spatial distributions of dye positions and structural heterogeneity of biomolecules. This toolkit in addition to modeling the structure of proteins—here HIV transcriptase with DNA—allowed the observation of multiple biomolecular conformations in solution with ultrahigh temporal resolution [198]. It thus appears as exceptional toolkit for quantitative dynamics structural information of biomolecules. Lastly amalgamation of the two worlds of SM mechanical manipulation and fluorescence holds enormous potential to reveal the correlation between conformational dynamics and mechanical properties [199,200].

In this review, we highlighted some of the insights provided by single molecule techniques on protein folding, conformational dynamics and function that are currently being recognized as interdependent events perceptible by the protein’s free energy landscape. The heights and the energy wells of the landscape define the pathway and energetics of folding to the native protein state while the amplitudes of the energy barrier and the thermodynamics between local minima dictate whether proteins rapidly interconvert between conformations or remain trapped in rarely sampled conformational and thus functional states. The existence of subpopulations of proteins with different functionality is anticipated to be crucial for cellular processes involving low copy numbers of proteins such as gene expression [201], signal initiation [20] phenotype switching [15] and even adaptive evolution [164] and is correlated to bacterial persistence to antibiotics [202]. Importantly, subpopulations might have subtle and yet significant differences in the affinities of their binding sites with regulatory cofactors and proteins partners that underlie their function and regulation. Single molecule functional and structural studies are ideally suited for directly observing their existence abundance and life times of these dynamics. Combining SM readouts with complementary interdisciplinary computational studies and spectrometric techniques such as NMR will enable bridging of multiple time scales and offer a holistic picture of protein dynamics and structure-function correlations. Integrating this knowledge into protein picture may contribute to the *in silico* design of novel pharmaceutics with increased efficiencies [203,204]. Similarly dynamic insights of both protein conformational ensembles, and the transition energy barriers could profoundly contribute in the “*de novo*” design of protein function [205,206] and engineering of enzymes with tailor made functionalities [131].
